# Implementing precision oncology in Latin America to improve patient outcomes: the status quo and a call to action for key stakeholders and decision-makers

**DOI:** 10.3332/ecancer.2024.1653

**Published:** 2024-01-08

**Authors:** Eva Maria Ruiz de Castilla, Maurice Mayrides, Haydée González, Francisco Vidangossy, Tatiana Corbeaux, Nancy Ortiz, Claudia Amaya, Alexandra Nuñez, Diego Fernando Jimbo Jimbo, Adela Ayensa, Mayra Galindo, Karla Ruiz, Juan Manuel Pérez

**Affiliations:** 1 Latin America Patients Academy, Miami, FL 33156, USA; 2 Linfomas Argentinas, Tucumán 731, Buenos Aires 1049, Argentina; 3 Fundación Cancer Vida, Santiago 8500000, Chile; 4 Oncoloop, Santiago 7501042, Chile; 5 Foro Nacional de Cancer Chile, Santiago 755000, Chile; 6 Fundacion SENOSama Bucaramanga, Santander 680002, Colombia; 7 Asociación Unidos Contra el Cáncer, San Jose 10103, Costa Rica; 8 Familias Unidas por las Enfermedades Catastróficas, Leopoldo Avado, Cuenca 010107 Ecuador; 9 Salvati, Calle Eugenia No 13 – 102, Col Nápoles, Benito Juarez, Ciudad de México 03810, México; 10 Asociación Mexicana de Lucha contra el Cáncer, Zacatecas No 24-4to piso, interior 404, Roma Nte, Cuauhtémoc, Ciudad de México 06700, México; 11 Calle Conde de la Monclova 363 of 306, San Isidro, Lima 15073, Peru; 12 Fundación Un Amigo como Tu, Ave Correa y Cidrón Esq Abraham Lincoln, Edif Profesionales Unidos, Suite 303, Santo Domingo, DN, Santo Domingo 10102, Dominican Republic

**Keywords:** precision oncology, precision medicine, Latin America

## Abstract

**Background:**

The advent of precision oncology (PO) has revolutionised diagnostic and follow up strategies and improved clinical outcomes for cancer patients. However, socio-economic inequalities in the level of implementation of PO in different countries is a prevailing issue. To improve this situation, the Latin America Patients Academy has gathered the recommendations of healthcare professionals and social civil members experienced in cancer management from Mexico, Guatemala, Costa Rica, Dominican Republic, Panama, Colombia, Chile, Ecuador, Peru and Argentina regarding the areas that need to be prioritised to improve the access to PO in Latin American (LATAM) countries.

**Methods:**

This manuscript is the culmination of a series of educational campaigns and panel discussion aimed at improving the implementations of PO in LATAM that took place from June 2021 to January 2022.

**The status of PO in Latin America:**

the level of PO implementation is generally low with some exceptions. The number of clinical trials and articles published with keywords related to PO from LATAM countries is drastically lower than in Europe and the United States. Despite sharing many complex challenges, progress is taking place in some countries in the region.

**Focus areas defined by the expert panel:**

The expert panel determined the areas of PO that should be improved by LATAM countries to improve its implementation through cancer care plans, educational programs and collaborative strategies. These initiatives should increase awareness about PO in the region and eventually improve cancer control in the region.

## Introduction

Cancer research used to be centred on drugs and tumour types. Yet, an improved understanding of genomic subgroup variability, tumour microenvironment, baseline patient and tumour characteristics, comorbidities, and other covariates has driven a paradigm shift in oncology that now better addresses population heterogeneity. The new approach is precision oncology (PO) which utilises gene-directed, histology-agnostic, and individualised approaches based on biomarker profiling to improve patient outcomes by directing the use of novel targeted agents such as small molecules, monoclonal antibodies and cell-based products [1].

As shown by three meta-analyses including over 83,000 patients from phase I and II studies, and studies leading to Food and Drug Administration drug approvals, the advent of PO with the use of personalised biomarker-based treatment strategies has indeed led to significantly improved efficacy and patient outcomes (overall survival, response rate, and progression-free survival), becoming independent predictors of better outcomes associated to lower treatment-related deaths across various malignancies [2–4]. Biomarker-based approaches incorporating biomarker status as a covariate have increased fivefold the likelihood of drug approval and clinical trial success rates for melanoma, breast and lung cancer studies [5].

Additionally, the more precise diagnoses achieved by identifying targetable cancer sub-types have improved clinical outcomes [6]. A further source of benefits to patients is precision medicine-enhanced oncologic follow up through targeted treatment response rates as it is becoming key to determining when cancers become resistant to a line of treatment and other treatment options should be considered [7].

Beyond efficacy and safety outcomes, PO has also helped overcome mechanisms of drug resistance and relapse and improved the quality of life of cancer patients allowing them to achieve milestones previously perceived as impossible [8]. Another benefit of PO to healthcare systems and patients is cost reduction since providing treatments to specifically identified populations increases the benefits for patients more likely to respond and minimises costs and side effects for those that would not respond [6].

Despite all the progress PO has catalysed, socio-economic inequality in this area of healthcare is a prevailing issue as shown by a systematic review including 38 studies and over a million patients, where low socio-economic status was associated with modestly lower biomarker testing and significantly lower targeted therapy utilisation [9]. In their review ‘Global inequities in precision medicine and molecular cancer research’, Drake *et al* [7] explain that although there are molecular profiling initiatives globally, most studies have been conducted in high-income countries. Thus, cancer patients in these countries are benefiting the most from PO.

Meanwhile, the molecular landscape from various cancers from low-middle income countries (LMICs), which differ geographically and by genetic ancestry from high-income countries, is not well known and good quality evidence is lacking. Consequently, as oncology becomes increasingly driven by molecular alterations and the burden of cancer rises in LMICs, these patients will suffer disproportionally from PO inequities [7]. This is certainly the case in Latin America (LATAM), where the widespread implementation of PO is hard to afford due to financial and technical challenges related to precarious healthcare systems and poor overall access to good quality healthcare [10].

LATAM’s fragmented healthcare, high poverty rates, and public/private healthcare disparities cause patients dependent on public healthcare settings to have difficulty accessing targeted therapies and the scarce laboratories in the region that perform molecular profiling [11]. In order to improve the current situation, the Latin America Patients Academy has gathered the recommendations of healthcare professionals and social civil members experienced in cancer management from Mexico, Guatemala, Costa Rica, Dominican Republic, Panama, Colombia, Chile, Ecuador, Peru and Argentina regarding the areas that need to be prioritised to improve PO in LATAM countries. This manuscript is the culmination of a series of educational campaigns and panel discussion aimed at improving the implementations of PO in LATAM. Its ultimate goal is to disseminate this call to action to improve genetic testing and the implementation of PO in LATAM.

## Methods

A panel constituted of PO specialists and patient representatives analysed and discussed key topics to advance PO initiatives and make it a priority within the regional healthcare systems. The types of cancer addressed were breast (and other gynecologic cancers), colon, lung and stomach.

Four meetings between PO specialists and patient representatives took place via the Zoom virtual meetings platform between June and September of 2021. They included educational sessions about current and newer PO testing techniques and drugs and a panel discussion about implementation problems and potential initiatives to improve cancer detection and PO treatment. A consensus statement with recommendations and a call for action was written based on these discussions. This was followed by an education campaign that took place between September 2021 and January 2022 aimed at educating patients and professionals about PO in LATAM. Educational materials, the consensus statement and letters were sent from the patient advocacy groups (PAGs) to stakeholders in each country.

In addition, PubMed searches were performed. The words ‘targeted’ and ‘molecular’ were chosen because when searched together with ‘cancer’ they generally lead to results involving targeted therapies and molecular profiling. PubMed was searched via the string (("[country]"[Title/Abstract]) AND (cancer[Title/Abstract])) AND (targeted/molecular[Title/Abstract]) for all types of publications from each of the listed countries.

Clinicaltrials.gov was also searched using the words ‘cancer’, ‘targeted’ and ‘molecular’ for all status studies, including both interventional and observational studies, and only those currently recruiting.

## Results and discussion

### The status of PO in Latin America

The level of PO implementation in LATAM is generally low except in Argentina, Chile and Costa Rica. Oncologists hesitate to prescribe targeted therapies because health insurance sometimes does not cover them. Biomarkers are only used to test special cases where there is a suspicion that there could be a mutation rather than to test all patients. Furthermore, most of the implementation of PO in LATAM is within the private part of the local healthcare systems. Public healthcare institutions in this region are not capable of covering these advanced testing techniques and therapies. In addition, the local treatment guidelines are not up to date nor regionally harmonised nor updated systematically. Thus, standards of care in each country are different and healthcare professionals lack evidence-based guideline support to be able to argue that using such therapies is an accepted treatment in their countries. Moreover, each country has a different situation due to a combination of factors.

**Figure 1.**
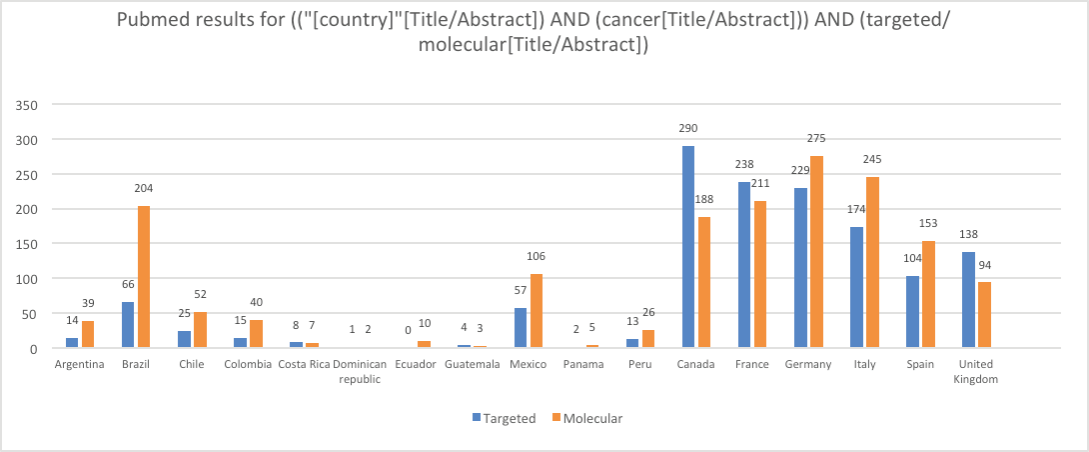
Results from PubMed searches by country for (("[country]"[Title/Abstract]) AND (cancer[Title/Abstract])) AND (targeted/molecular[Title/Abstract]). The values for the US are not included in the chart because they impair the visibility of the other values; for the terms ‘targeted’ and ‘molecular’ the values for the US are 1,674 and 1,835, respectively.

Methodic clinicaltrials.gov and PubMed searches provide a high-level and objective perspective of the current level of PO implementation in different LATAM countries compared to European countries and the United States (US). The words ‘targeted’ and ‘molecular’ were chosen because when searched together with ‘cancer’ they generally lead to results involving targeted therapies and molecular profiling. Clinicaltrials.gov was searched for ‘cancer’ and ‘targeted’ and ‘molecular’ keywords for all status studies and those only recruiting. As Table 1 shows, the difference in the number of studies regardless of their status and those currently recruiting between representative LATAM countries and European countries, and the US is very large. For example, the number of studies with the words ‘cancer’ and ‘molecular’ currently recruiting in LATAM countries is in the tens, while in European countries it is in the hundreds, and in the US, it is in the thousands. Similar results were obtained when PubMed was searched via the string (("[country]"[Title/Abstract]) AND (cancer[Title/Abstract])) AND (targeted/molecular[Title/Abstract]) for publications from each of the listed countries (Figure 1). Although there may be some studies that are not on cancer or were ‘targeted’ or ‘molecular’ may not refer specifically to targeted therapies and molecular profiling, these searches provide an overall impression of the contrast between LATAM, European countries, and the US.

LATAM countries share common challenges such as political instability, economic uncertainty, precision medicine focused only in rare diseases, inability to take advantage from specialised and advanced medical technology, geographic limitations that put rural areas at a disadvantage, the preconception that PO costs are inherently higher, the need for very sophisticated molecular testing techniques, unequal access to targeted therapies, slow incorporation of new technologies, lack of healthcare professionals specialised on PO, and healthcare professionals concentrated around large cities. Lack of disease-specific patient education and patient support in general hinder the ability of patients to advocate for themselves by inquiring about PO treatment possibilities. All these problems contribute to the widespread difficulty accessing biomarker testing, targeted therapies, and patient education in this region.

Funding to facilitate access to PO treatments is another issue that LATAM countries face [10]. The cost and affordability of oncologic drugs compounds this problem because costs tend to be higher in LMICS than in high income countries (HICs), and there is often no relationship between drug prices in LMICs and their gross domestic products [12,13]. As reported recently by Moye-Holz and Vogler [14] in their study comparing the prices and affordability of oncology drugs (bevacizumab, cetuximab, dasatinib, everolimus, imatinib, mercaptopurine, nilotinib, panitimumab, pazopanib, rituximab, sorafenib, sunitinib, trastuzumab) in 16 countries in Europe (Austria, France, Germany, Greece, Hungary, the Netherlands, Poland, Romania, Spain, Sweden and the UK) and LATAM (Brazil, Chile, Colombia, Mexico and Peru): upper-HICs (UHICs) in Europe had lower adjusted prices for PO drugs than LATAM and LHICs in Europe. The cost of the drugs were also less variable in UHICs than in LHICs and LATAM. Accordingly, the affordability assessment the authors performed showed that PO drugs were more affordable in HICs than in LATAM and other middle income European countries. Some of the reasons they propose for the higher and variable PO prices in LATAM include lack of pricing and reimbursement regulations and the lack of bargaining power of these countries [14]. As minuciously described in the supplementary tables of their manuscript, mercaptopurine 50 mg, trastuzumab 150 mg, imatinib 100 mg, and imatinib 400 mg were the most affordable drugs in all countries. The drugs bevacizumab 100 mg, bevacizumab 400 mg, cetuximab 100 mg, dasatinib 50 mg, everolimus 5 mg, panitumumab 100 mg, sunitinib 12.5 mg, and trastuzumab 440 mg were the least affordable [14]. In terms of costs per country, for most drugs, Sweden and the UK had the lowest prices, and drug cost in Poland and Romania were some of the highest. Peru and Brazil had the highest prices for some drugs [14]. Another notable reason for increased PO drug costs in LATAM’s countries are frequent economic crises with out-of-control inflation as highlighted recently in The Lancet Oncology [15]. Research funding is another problem: as a review by members of the Latin American (LATAM) Cooperative Oncology Group and other LATAM institutions explained, governmental grants in LATAM are insufficient to maintain competitive research initiatives. Investigator-initiated research is funded mostly by pharmaceutical companies, government programs, fundraising campaigns and self-funding by investigators or institutions. Thus, foreign sources largely dictate LATAM’s research agenda due to scarce local funding [16]. To improve the affordability of PO drugs, these should be better represented in the World Health Organization Model List of Essential Medicines (WEM) list. According to Bharadwaj *et al* [17] as of 2019 the WEM list only included eight targeted therapies: rituximab, trastuzumab, imatinib and erlotinib among others. This list should be reviewed to include newer drugs with greater efficacy than the ones currently included. However, drugs with the same efficacy and cost as the current drugs should also be added to broaden the range of PO drugs. Lastly, drugs that treat cancers not represented by the currently included drugs should be added, as well. Adding biosimilars onto the WEM list could help improve access [17]. Also, the Access to Oncology Medicines Coalition (Coalition), an initiative from the Union for International Cancer Control and more than 40 international organisations, aims to address barriers to availability, affordability, and appropriate use of oncology drugs in LMICs. Some of its strategies include increasing the availability of World Health Organisation Model List of Essential Medicines (WEMs), generics, and biosimilar cancer drugs and increasing the number of patented cancer medicines and new medicines in the pipeline [18].

Finally, patients cannot benefit from PO if they do not have access to biomarker testing and genomic sequencing to determine their eligibility for PO treatments in the first place [19]. As explained in the review by Alvarez-Gomez *et al* [20] LATAM’s countries face challenges in genomic sequencing: sequencing platform upgrades have prohibitive costs in these countries making genomic cancer projects hard to sustain and worsening PO access inequality. Moreover, the cost of genomic sequencing in general makes PO treatments unaffordable in the region as minimal testing for standard-of-care treatments is often not covered and healthcare budgets for PO are scarce [21]. Another factor is the region’s ethnic and genomic heterogeneity; this represents a challenge in itself and an opportunity, as it can help optimise the limited resources available [16, 20]. Among the potential solutions to improve access to genetic testing mentioned by Alvarez-Gomez *et al* [20] efforts to redirect genomic sequencing towards cancers that impact public health the most in the region is paramount. Health policies should guarantee accurate diagnosis and access to treatments independently of their cost. Multidisciplinary teams and cancer research networks should be strengthened. Training is also important for genomic sequencing to be widely adopted and used; but preventing the emigration of highly specialised oncology and pathology specialists from LATAM is critical [20]. Additionally, the LATAM Cancer Research Network (LACRN) has highlighted the importance of sustained economic contributions from local governments to implement genomic research in LATAM [22].

Despite all these obstacles, some progress is taking place. Argentina has taken the lead with ongoing initiatives to encourage the implementation of precision medicine at provincial and national levels, these include: generating a genomics biobank, sequencing and analysing exons of patients with rare diseases, developing a panel of precision genomic oncology, and others [23]. In Colombia, a new private cancer treatment centre was opened recently. In Brazil, a new law that improves access to liquid biopsies and treatments for oral cancers was enacted recently. In Peru, another law is helping improve disease-specific education but there are still access problems and patients must pay for PO out of pocket. Pharmaceutical companies provide some support by paying for geniting testing. Medical societies and private clinics across the region are implementing PO. In the last 3–4 years, new biomarker testing companies have been established locally. According to Venezuelan experts, several groups are collaborating to improve oncology clinical research in LATAM: the LATAM Cooperative Oncology Group, the US-LACRN, the LATAM Federation of Cancer Societies, the LATAM Consortium for Lung Cancer Research (CLICaP), and the Ibero-American network of Pharmacogenetics and Pharmacogenomics [10]. Some of their findings include the fact that there is considerable heterogeneity in epidermal growth factor receptor (EGFR) and Kirsten rat sarcoma viral oncogene homologue (KRAS) mutations between LATAM populations: [24, 25] EGFR mutation frequency was found to be 26.4% but it varies according to the population. The EGFR mutation frequency was higher in Peru than in Argentina, which could be attributed to ethnic differences. Peru has received significant Asian migration, where the EGFR mutations occur in 30%–50%, while Argentina has received mostly European migration, whose EGFR mutation rate is 8%–13% [25].

### Focus areas defined by the expert panel and discussion

The following three focus areas resulted from the discussions between PO specialists and patient representatives from the participating countries. They provide recommendations for action plans that prioritise the access to PO in LATAM as the experts agree that to reach this goal it is necessary to improve PO readiness of LATAM’s healthcare systems.

The group recognised many intervening challenges. The barriers to the widespread adoption of PO in LMICs are numerous, complex, and multifactorial and the initial focus areas herein aim to persuade local governments that PO is an important strategy for more effective cancer control at the national level.

### Cancer control plans

- Governmental policies that include PO: these official policies supported by scientific evidence and experts in the field are the main mechanism to guide national cancer control strategies. Few countries have cancer control plans that include PO because most were developed before the advent of PO. Some laws mandate periodic updates in these plans, but in other cases governments will have to be encouraged to update them. They must be updated periodically to incorporate the adoption of PO at national levels.
- Medical specialists and patient representatives to contribute to strategic plans that include PO: in general, there is enough experience in medical specialties in each country to elaborate strategies and national cancer institutes can summon meetings to this end. Patient representatives can also lead and collaborate in these efforts.
- Ensure PO policies exist in all countries: the countries that have higher PO implementation have local policies/laws. Countries without these laws in place should advocate for higher commitment to advancing PO locally.
- Assess unmet needs on genomic sequencing specific to PO: since some general national plans around genetic mapping and detection are not specific enough for oncology, the needs of each country will have to be assessed to identify unmet needs and opportunities.
- Promote compliance with clinical practice guidelines that include POI: The recently published clinical practice guidelines developed by local medical societies for different cancer types and focused on PO can help inform cancer control strategies or serve as foundation for these strategies.
- Elaborate local plans based on countries that have succeeded implementing PO: examples from other countries or regions can be used as models to elaborate local plans.

### Establishment of educational initiatives

- Evidence-based policymaking: the main objective is to generate political will supported by medical, economic and quality-of-life data.
- Educational initiatives led by independent organisations: PAG involvement in educational initiatives is critical as objective parties focused on improving patient health without economic conflicts of interest. If consulting organisations are used to study and make recommendations on these subjects, they should be independent from political interests.
- Detailed economic evaluations of PO investments: patient representatives should consider economic evaluations to estimate the cost of necessary investments in PO along with cost-benefit analyses and budgetary impacts. These should be performed by qualified economists able to present this information clearly and accurately to policy decision-makers.
- Implementation research to define challenges and propose solutions: patients and their representatives must be clear and specific in their demands to policymakers. They should develop lists of implementation challenges and potential solutions to consider. Implementation research can help inform these efforts.
- Educate decision- and policymakers about PO: healthcare decision- and policymakers should be informed about PO, especially about its benefits, the existing challenges, and the best ways to adopt it and implement it.
- Effective PO communicators: identify and recruit effective medically/scientifically trained communicators to lead educational and dissemination events.
- Specific PO objectives: decision-makers should focus on specific objectives such as new cancer control plans, new laws and new coordinating committees.
- Draw from other countries’ experiences: the experience of countries that have successfully implemented or are planning to implement PO can be helpful in identifying lessons learned and critical implementation points.

### Collaborative strategies

- Common agendas focused on PO: PAGs should develop a common policy agenda clearly defining the journey for PO access to benefit cancer patients the most.
- Identify and troubleshoot challenges to PO research: the scientific community should identify and document the obstacles to conduct clinical trials and other research projects that are critical to PO implementation. These challenges must be faced and potential solutions to them should be considered country-by-country.
- Clinical guidelines develop through collaboration: develop clinical guidelines with updated scientific PO data in a collaborative manner to enhance healthcare professional training.
- Support and investments from the biopharmaceutical industry to improve PO in each country: the payment of molecular testing for individual patients related to specific targeted therapies is not sustainable and it is one of the main reasons for disagreements between the industry, healthcare professionals and governments.
- Collaborations between industry and healthcare systems: industry coalitions should discuss the role of their members in PO for public healthcare systems including access plans for specific tests and treatments.
- Private and public sector collaborations: private laboratories and other businesses that benefit from PO should recognise the value of widespread PO adoption and share resources and knowledge needed for implementation in the public sector.
- International organisation’s involvement in local PO: multinational institutions could be engaged to finance the construction of PO infrastructure in rural and/or poor regions.
- Harmonise laboratory standards and establish training programs: create collaborative alliances between international pathology and molecular analysis teams and health authorities to harmonise laboratory standards and to establish training programs for the next generation of laboratory professionals. These alliances should help analyse and fill the gaps in human resources capabilities required to implement PO.
- PO expert, PAG and regulatory authorities’ collaboration: the collaboration of PO experts, patient advocates, and regulatory authorities is indispensable to review molecular testing, detection technologies and targeted therapies to obtain approval, financing and dissemination within healthcare systems.
- Industry, private and public sector collaborations: promote discussions about the industry’s role in PO’s implementation in the public sector including agreements about access to specific tests or treatments. The private sector (which benefits from PO) should recognise the value of closer associations with the public sector that promote sharing resources and PO knowledge.
- Informed decision-makers: ensure that leaders and health technology evaluators have comprehensive knowledge of PO ecosystems before considering new technologies.
- Use Pan American Health Organization’s (PAHO’s) resources related to PO: the pertinent authorities in each country should consider reviewing the applicability of the four policies proposed in the document ‘Access and rational use of strategic and high-cost medicines and other health technologies’ from PAHO.

In their practical roadmap for action on PO, Baird *et al* [26] described the rationale for a PO approach that unites all the healthcare stakeholders to encourage a collaborative and patient-centred methodology like that proposed by our expert panel. They also mention several important initiatives from the last 2 years: Europe's Beating Cancer Plan; the European Union Mission on Cancer; no one missed, by the US LUNGevity organisation; initiatives in lung cancer care; the US Cancer Moonshot which was relaunched in 2022. These initiatives are to foster advances in PO implementation, but that can only happen within healthcare systems and governments that also promote and adopt advances. Thus, they also encourage an ‘intersectoral multistakeholder approach’ where public and private stakeholders collaborate to integrate their PO strategies and solutions. They explain that PO can deliver better patient outcomes by focusing collaboration on three main activities: data and evidence development about PO benefits on patient outcomes; education and tools for patients, clinicians and other stakeholders to support informed decision-making; and addressing access barriers in the patient pathway. In their practical roadmap for precision medicine advancement, they mention ‘Patient Focused Medicines Development’ which was established in 2015 to embed meaningful patient engagement throughout the continuum of drug development and ‘from testing to targeted treatments’ (FT3), which was established in 2020 from common experiences of success and failures and a shared commitment to make precision medicine mainstream [26].

Mateo *et al* [27] recently shared their perspective on steps to resolve the main challenges to the implementation of PO, these include items covered by our expert panel, for instance: democratise access to genomics testing, ensuring good-quality clinical trials provide robust evidence for new drugs and technologies, educate physicians to interpret genomic data, and empower patients to be able to participate in decision-making about their conditions. They also suggest a multi-stakeholder approach to generate evidence, value assessments and healthcare delivery for PO to benefit patients [27].

Precision medicine’s incorporation into healthcare systems has been challenging partly because the body of research was under development and the technologies surrounding it were lacking. However, as Doxzen and Bowman [28] in their World Economic Forum article explained, there has also been a lack of coordination. To improve in this aspect, they suggest that policy-makers can implement agile processes to advance precision medicine and improve implementation processes. First, they can engage the industry to ensure that innovations comply with regulatory agencies’ standards. Second, they can incorporate the public into the research process. Third, governments should take a holistic perspective, monitoring each step in the implementation of PO to identify weaknesses and protect against disruptions in the pipeline. Lastly, governments should continually evaluate the balance between innovations and risks through periodic audits and communication with all stakeholders. Improvements based on key lessons learned can help policymakers keep up with rapid technological advancements [28].

Regarding genomic sequencing and the diagnostic technologies linked to PO, the European Cancer Patient Coalition, the International Quality Network for Pathology and the European Federation of Pharmaceutical Industries and Associations recently published short- and long-term policy recommendations to improve biomarker testing access in Europe. The short-term recommendations included: parallel approval of the medicine and associated biomarker testing, a national system for biomarker test value assessment, dedicated biomarker test budgets, mandatory International Organisation for Standardisation accreditation and External Quality Assurance scheme participation, regional testing centres, stakeholder education, and centralised data collection. The long-term recommendations included: harmonised approaches along the test development continuum, including guidance on biomarker use during clinical trials and test value assessment, centralised testing infrastructure, data sharing and guidelines on comprehensive testing [19].

One of the weaknesses of this expert panel’s work is that it has not covered the role of artificial intelligence (AI) specifically. But in their review about the integration of AI to PO in LATAM, Sussman *et al* [29] explain how AI can help manage and process the vast amounts of patient data generated by all the omics and help find associations between multiple variables. In PO, AI could be used to accomplish tasks that human experts would normally perform. It can be particularly helpful in digital pathology, interpreting imaging tests, molecular profiling, germline variant discovery, radiomics and radio genomics. Although AI has promising applications in PO and it could bridge unmet needs in the region such as insufficient health providers, to be able to implement it, some healthcare providers will have to be trained and qualified on AI nevertheless [29].

## Conclusion

As stated in The Lancet’s editorial ‘20 years of precision medicine in oncology’, a highly competitive market and pharmaceutical companies’ initiatives put a focus on technological breakthroughs rather than in health policies. Consequently, insufficient research into the implementation of precision medicine opened a gap between innovations and improved patient outcomes globally [30].

This expert panel determined the areas of PO that should first be improved by LATAM countries to optimise its implementation through cancer care plans, educational programs and collaborative strategies. Stakeholders at all levels have to work for healthcare systems to integrate molecular profiling into oncologic pathways and ensure that patients of all socio-economic levels have access to PO [8].

This group of experts and patient representatives concur that if some of the initiatives previously enumerated could be initiated in each of the focus areas while considering the difficult economic and health situations of LATAM countries, the first steps to improve PO access for cancer patients and healthcare systems will be taken. At the very least, they will create awareness about the challenging but valuable path to improve cancer control in the region.

Future initiatives should delve more deeply into how to improve the use of genetic testing and big data to use existing resources more efficiently in LATAM.

## Conflicts of interest

The author(s) declare that they have no conflict of interest.

## Funding

Funding support for this initiative was provided by AstraZeneca.

## Figures and Tables

**Table 1.** Results of clinicaltrials.gov searches by country for the terms cancer, and targeted/molecular for recruiting all status studies.

	Recruiting		All statuses	
	Targeted	Molecular	Targeted	Molecular
Argentina	24	98	248	540
Brazil	35	140	349	790
Chile	23	68	155	309
Colombia	14	24	83	166
Costa Rica	4	9	15	27
Dominican Republic	2	1	3	5
Ecuador	0	0	11	12
Guatemala	8	11	33	46
Mexico	23	105	239	513
Panama	3	4	33	47
Peru	8	26	117	230
Canada	200	417	1,291	2,796
France	331	602	1,688	3,291
Germany	150	383	1,161	2,530
Italy	168	436	1,177	2,653
Spain	203	477	1,309	2,562
UK	183	354	1,200	2,358
US	1,426	2,685	7,729	17,673
